# Gut microbiome changes and cancer immunotherapy outcomes associated with dietary interventions: a systematic review of preclinical and clinical evidence

**DOI:** 10.1186/s12967-025-06586-0

**Published:** 2025-07-08

**Authors:** Csenge Somodi, David Dora, Mátyás Horváth, Gabor Szegvari, Zoltan Lohinai

**Affiliations:** 1 Translational Medicine Institute, Semmelweis University, Tűzoltó Utca 37-47, 1094 Budapest, Hungary; 2 Department of Anatomy, Histology, and Embryology, Semmelweis University, Budapest, Hungary

**Keywords:** Gut microbiome, Cancer, Immunotherapy, Diet, Fiber intake, Ketogenic diet

## Abstract

**Introduction:**

Cancer patient’s survival has gradually improved due to immune checkpoint inhibitors (ICIs). Several studies showed a possible association between the intestinal microbiome and ICI efficacy. Strategies for modifying the composition of the gut microbiome encompass various dietary interventions, which may have distinct impacts on the outcomes of ICI-treated patients. In our systematic review, we explored how dietary habits correlate with therapeutic responses in cancer patients and cancer mouse models undergoing immunotherapy.

**Methods:**

A systematic review was conducted using search terms: “cancer”, “immunotherapy”, “diet”, and “microbiome”, from Medline, Web of Science, Scopus, and Cochrane Library databases. The outcomes in the clinical studies were overall response rate (ORR), overall survival (OS), or progression-free survival (PFS) in human studies. In mouse studies, change in tumor size was the endpoint. The comparator attributions were questionnaire-based dietary interventions.

**Results:**

Nineteen articles met the inclusion criteria and were included in the review (6 prospective cohort studies, 1 cross-sectional observational study, and 12 mouse studies). A consistent association was observed between high (vs. low) fiber consumption and improved therapeutic response with a pooled odds ratio of 5.79 when including all human prospective cohort studies. In mice, limited availability of methionine, cysteine, and low intake of leucine and glutamine was linked to reduced tumor progression. Combining ICIs with intermittent fasting or a fasting-mimicking diet significantly decreased tumor volume in mouse melanoma models. In humans, a higher relative abundance of short-chain fatty acid (SCFA) and lactic acid-producing bacteria—particularly Faecalibacterium prausnitzii and Akkermansia muciniphila—correlated with objective response rates (ORR). Similar microbiome alterations were observed in mouse models. Increased fiber intake enhanced ICI efficacy in mice by modulating the gut microbiome, primarily via elevated SCFA production—an effect also reflected in human studies.

**Conclusion:**

Intermittent fasting, high fiber, and low sugar consumption are significantly associated with better ICI outcomes. The studies revealed alterations in microbiota composition linked to diet, and these findings were confirmed in animal models, regarding the production of SCFAs and lactic acid, as well as an increase in Bacteroidota/Bacillota ratio and microbial diversity.

**Supplementary Information:**

The online version contains supplementary material available at 10.1186/s12967-025-06586-0.

## Introduction

The diet-related gut microbiota changes might affect ICI efficacy, and there can be differences between the microbial communities of responders and non-responders. Cancer immunotherapy represents a significant advancement in patient outcomes in oncology. Various cancers have responded to immunotherapy, albeit with a need to understand the underlying mechanisms [1]. Despite its potential, there is a requirement for improving the response rate of immune checkpoint inhibition (ICI), which is currently at 15–20% in good performance status [2].

Bacteria densely inhabit the intestines and continuously interact with the immune system. The combined genetic material of bacteria and other microorganisms in this ecological community has been the subject of growing scientific interest over the last twenty years [3]. The gut microbiota also serves as a host metabolism regulator, while influencing the appropriate maturation and operation of the immune system [4]. The anti-cancer immune response might be modulated by gut microbiota through antitumor CD8 T-cell or macrophage maturation in the gut [5, 6]. Nonetheless, disruption of the intestinal microbiota due to external factors (e.g. dietary habits, antibiotic consumption), or immune system alterations may lead to widespread dissemination of detrimental microorganisms, increased vulnerability to harmful microbial infiltration, and irregular immune reactions [3, 7]. Growing evidence corroborates the involvement of the microbiome in the efficacy of cancer treatment, as evidenced by several recent studies highlighting the impact of the intestinal microbiome, particularly on the effectiveness of ICI in various forms of cancer, including melanoma, lung, and renal cancer [8–13].

Several bacterial species have been associated with improved immune checkpoint inhibitor (ICI) responses. Akkermansia muciniphila has been shown to enhance ICI efficacy by stimulating cytokine secretion via MHC Class II-restricted CD4+T cells and dendritic cells, as reported in studies by Routy et al. [9] and Lu et al. [14]. Certain Bacteroides species, including B. thetaiotaomicron and B. fragilis, have been linked to the induction of TH1 immune responses and dendritic cell maturation in tumor-draining lymph nodes, as demonstrated by earlier [15, 16]. Bifidobacterium species, such as B. breve and B. longum, have been associated with increased accumulation of antigen-specific CD8+tumor-infiltrating lymphocytes (TILs) and MHC Class II dendritic cells, as observed in multiple studies [4, 17]. Members of the Ruminococcaceae family, including R. obeum and R. bromii, have been shown to elevate levels of CD4+ and CD8+T cells, enhancing anti-tumor immunity [9, 18]. Additionally, Faecalibacterium prausnitzii has been identified as a beneficial gut bacterium in ICI responders, potentially playing a role in immunomodulation, according to findings by Frankel et al. [16] and Botticelli et al. [19].

Alterations in gut microbiota might enhance ICI-efficacy, however, less is known about dietary interventions. More data is available connected to prebiotics or probiotics that have the potential to modify gastrointestinal microbiome composition [15]. Recent research has identified associations between favorable outcomes and diets such as ketogenic, plant-based, and microbiome-supporting regimens [16]. Ongoing clinical trials investigate the impact of dietary modifications, such as enhanced fiber consumption or personalized dietary interventions, on the microbiome’s composition and its corresponding clinical consequences. An increasing array of data concerning probiotics and shifts in microbial composition might influence the efficacy of immunotherapeutic methods; still, it is vital to establish particular dietary alterations associated with changes in the microbiome and beneficial microbial entities [15].

Even though different dietary patterns -and in parallel- different microbial communities can be observed in cancer ICI responder vs. non-responder patients; the mechanistic relations remain unclear. Analysis of pre-clinical studies along with clinical trial data evaluation might help to understand the underlying biology and complex interactions between microbiota and ICI efficacy. In this systematic review, we aimed to compile the current preclinical and clinical data on nutritional factors, associated gut microbiome taxonomy, and functionality for ICI efficacy.

## Methods

The systematic search was conducted according to the principles established by the Preferred Reporting Items for Systematic Reviews and Meta-analyses (PRISMA) statement [17].

### Study selection

To obtain relevant publications, the scanning of Scopus, Cochrane Library, Web of Science and Medline (through the Pubmed interface) was conducted and completed in 30 March 2024. The inquiry consisted of the following terms: “diet”, “cancer”, “immunotherapy”, “microbiome”, along with their corresponding terms and connections consolidated utilizing the boolean operator OR, succeeded by multiple limitations employing the AND operator. The exact search key can be found in Supplementary file 1. The query hits generated were archived in RIS format and subsequently brought into the abstract management tool Rayyan [18]. Research articles meeting the criteria for full-text examination were acquired via the subscription of Semmelweis University and preserved within Rayyan.

### Inclusion criteria

According to PICO framework, we posed the subsequent question: What are the principal dietary components that, in conjunction with the intestinal microbiome, may impact the results of immunotherapy? Based on the aforementioned scope of the study, the following inclusion criteria were prepared:

(I) Diagnosis of solid cancer patients (early and advanced-stage)
(II) Cancer Patients treated with immune-checkpoint blockade (anti-PD1, anti-PD-L1 and anti-CTLA4 or other ICI drugs included; neoadjuvant, adjuvant and advanced stage IIIB-IV treatments included; chemotherapy (CHT) + ICI, chemo-radiation (CRT) + ICI combinations are also allowed). Inclusion in clinical trials is permitted. The outcomes were reported in terms of response rate, overall response rate (ORR), progression-free survival (PFS), or overall survival (OS).
(III) Microbiome measured in stool samples
(IV) Microbial data performed by shotgun metagenomics or 16S RNA-sequencing
(V) Any nutrition or diet-related information available such as interventions or questionnaires were allowed
(VI) Mouse or human studies were allowed

### Exclusion criteria

(I) Reviews, systematic reviews, meta-analyses, editorials, correspondences, case series, case reports, commentaries, letters to editor, protocols, and conference abstracts
(II) Cancer vaccination, vaccines
(III) Exclusively in silico studies on open-access data
(IV) Primarily probiotic treatment without dietary information
(V) Specific plant extracts as intervention or unknown composition of the nutritional intervention
(VI) Purely in vitro (cell line, cell culture) or ex vivo (organoid) studies

### Manual selection

Two researchers (GSz and CsS) independently evaluated the abstracts according to the predetermined inclusion/exclusion criteria using a three-tiered (include/exclude/maybe) rating system. Should any disparities arise in the evaluation, the publication was subjected to a comprehensive full-text examination. During the full-text assessment, the same researchers scrutinized the publications with unrestricted access to information.

### Data extraction

The consecutive information was extracted from the studies: first author name, publication year, population, tumor type, treatment, observed dietary habit, type of the available microbial data, and outcome (Table 1). In studies where the mentioned data was not assessed, we used the not assessed (NA) sign in the tables. Additional information was obtained to provide a comprehensive summary of the results of the studies (Supplementary Table 1) Information on the comparision of the response and microbial alterations in association with the different dietary interventions, alpha and beta diversity measures, Firmicutes/ Bacteroidetes ratios (F/B ratio) (Table 2), and increased and decreased abundance of certain bacterial taxa after treatment (Table 3) were extracted from articles.

**Table 1:** Summary of included studies

Study	Year	Population	Type of study	Tumor type	Treatment	Observed diet	Microbial data	Outcome
Bolte et al.	[19]	Human	Prospective cohort study	Melanoma	Ipilimumab-nivolumab combi treatment (anti PD-1) + (anti CTLA-4), or (anti PD-1/anti PD-L1) + (anti CTLA-4)	Mediterranean diet	–	ORR, PFS at 12 months
Pietrzak et al.	[36]	Human	Prospective cohort study	Melanoma	Nivolumab or pembrolizumab (anti PD-1)	Overall habits (protein, fiber comsumption etc..)	NGS	ORR
Simpson et al.	[21]	Human	Prospective cohort study	Melanoma	Ipilimumab and nivolumab combi (anti PD-1) + (anti CTLA-4)	Overall habits (protein, fiber comsumption etc..)	–	Response rate
Spencer et al.	[20]	Human	Prospective cohort study	Melanoma	Anti-CTLA4 + anti-PD-1 combi, or anti-PD-1 monotherapy	Fiber intake	Metagenomics	ORR (responder: CR, PR, SD: PFS >= 6 months)
Nomura et al.	[34]	Human	Observational cross-sectional study	Solid tumors	Pembrolizumab, nivolumab	Overall habits (protein, fiber comsumption etc..)	–	ORR, PFS
Golčić et al.	[24]	Human	Experimental randomised animal study	Melanoma	Pembrolizumab, nivolumab, or nivolumab + ipilimumab	Overall habits (pro-inflammatory and anti-inflammatory etc...)	Metagenomics	ORR
Ferrere et al.	[27]	Mouse	Experimental randomised animal study	Melanoma	Anti-CTLA4 + anti-PD-1 combi or anti-CTLA4/ anti-PD-1 monotherapy	Ketogenic diet	–	Tumor size growth
Boucher et al.	[25]	Mouse	Experimental randomised animal study	Melanoma, fibrosarcoma, colorectal cancer	Anti-PD-1	Inulin-enriched diet	16S rRNA sequencing	Tumor size growth
Han et al.	[28]	Mouse	Experimental randomised animal study	Colon carcinoma	Anti-PD-1	Inulin-enriched diet	16S rRNA sequencing	Tumor size growth
Zhang et al.	[1]	Mouse	Experimental randomised animal study	Colon carcinoma	Anti-PD-1	The effect of FMT and pectin-enriched diet	–	Tumor size growth
Ji et al.	[52]	Mouse	Experimental randomised animal study	Colon carcinoma	Anti-PD-1	Methionine-enriched diet	16S rRNA sequencing, metatranscriptomics	Tumor size growth
Kim et al.	[45]	Mouse	Experimental randomised animal study	Glioma	Anti-PD-1	High glucose diet	16S rRNA sequencing	Tumor size growth
Udumula et al.	[2]	Mouse	Experimental randomised animal study	Epithelial ovarian cancer	Anti-PD-1	Intermittent fasting	–	Tumor size growth
Tanaka et al.	[22]	Human	Prospective cohort study	Lung cancer	Anti-PD-1 (pembrolizumab)	Overall habits (protein, fiber comsumption etc..)	–	OS > 3 years, OS < 3 years
Cortellino et al.	[63]	Mouse	Experimental randomised animal study	Melanoma, lung cancer	Anti-PD-1, anti-PD-1, anti-PD-L1, anti-CTLA4	Fasting	–	Tumor size growth
Calderón-Montaño et al.	[53]	Mouse	Experimental randomised animal study	Renal cell carcinoma	Anti-PD-1	Vitamin, mineral and amino-acid enriched diets	–	Overall survival (developed symptoms of advanced disease and were sacrificed x days after tumor cell injection)
Kuehm et al.	[64]	Mouse	Experimental randomised animal study	Melanoma, colon carcinoma	Anti-PD-1, anti-CTLA-4, anti-LAG-3	Western and fructose enriched diet	Taxon specific sequencing PCR	Tumor size growth
Lam et al.	[65]	Mouse	Experimental randomised animal study	Lymphoma, colon carcinoma, melanoma	Anti-PD-1, anti-PD-L1	Western and pectin enriched diet	16S rRNA sequencing	Tumor size growth
Li et al.	[66]	Mouse	Experimental randomised animal study	Melanoma, colon carcinoma	Anti-PD-1	Inulin and mucin enriched diet	16S rRNA sequencing	Tumor size growth

**Table 2:** Diversity metrics and F/B ratio identified in the included studies, categorized by ICI-responder groups

Study	Alpha-diversity in responders/in the treatment group	Beta-diversity in responders/in the treatment group	Firmicutes/Bacteroides ratio in responders
Bolte et al. [19]	NA	NA	NA
Pietrzak et al. [36]	Decreased	Altered	Increased
Simpson et al. [21]	Not different	NA	NA
Spencer et al. [20]	Not different	NA	NA
Nomura et al. [34]	NA	NA	NA
Golčić et al. [24]	No signifincant difference between early and late responders	Increased in late responders	NA
Ferrere et al. [27]	NA	Altered	NA
Boucher et al. [25]	Altered	Altered	NA
Han et al. [28]	NA	NA	NA
Zhang et al. [1]	Increased	Altered	Increased
Ji et al. [52]	NA	NA	Increased
Kim et al. [45]	NA	NA	NA
Udumula et al. [2]	NA	NA	NA
Tanaka et al. [22]	NA	NA	NA
Cortellino et al. [63]	NA	NA	NA
Calderón-Montaño et al. [53]	NA	NA	NA
Kuehm et al. [64]	NA	NA	NA
Lam et al. [65]	NA	NA	NA
Li et al. [66]	NA	NA	NA

**Table 3:** Differentially abundant bacterial taxa in the included studies categorized by ICI-responder groups

Study	Increased abundance in responders	Decreased abundance in responders	Increased abundance in non-responders	Decreased abundance in non-responders	Increased abundance in treated	Decreased abundance in treated
Bolte et al. [19]	NA	NA	NA	NA	NA	NA
Pietrzak et al. [36]	Prevotella copri, Bacteroides uniformis	NA	Faecalibacterium prausnitzii, Desulfovibrio intestinalis	NA	NA	NA
Simpson et al. [21]	Faecalibacterium prausnitzii, Butyricicoccus pullicaecorum, Akkermansia muciniphilia	NA	NA	Methanogenic archaea, Akkermansia muciniphilia, and Ruminococcaceae	NA	NA
Spencer et al. [20]	Ruminococcaceae	NA	NA	NA	NA	NA
Nomura et al. [34]	NA	NA	NA	NA	NA	NA
Golčić et al. [24]	Higher abundance of Prevotellaceae in early responders	NA	NA	NA	NA	NA
Ferrere et al. [27]	NA	NA	NA	NA	Akkermansia muciniphila, Ruthenibacterium lactatiformans, Pseudoflavonifractor capillosus, Eisenbergiella massiliensis	Clostridium asparagiforme, Lactobacillus caviae, Lactobacillus taiwanensis, Lactobacillus reuteri, Lactobacillus gasseri, Lactobacillus hominis, Lactobacillaceae
Boucher et al. [25]	NA	NA	NA	NA	Bifidobacterium (Actinobacteriota), Bifidobacterium animalis subsp. animalis	Clostridium saudiense, Clostridium disporicum, and Clostridium celatum (Firmicutes)
Han et al. [28]	Akkermansia, Lactobacillus, Roseburia	Oscillibater	NA	NA	NA	NA
Zhang et al. [1]	Ruminococcaceae, Faecalibacterium, and Holdemania	NA	NA	NA	Lactobacillaceae, Bifidobacteriaceae, Erysipelotrichaceae, and Ruminococcaceae	NA
Ji et al. [52]	NA	NA	NA	NA	NA	NA
Kim et al. [45]	NA	NA	NA	NA	Erysipelotrichaceae, Desulfovibrionaceae, AC160630_f, Rikenellaceae, Odoribacteraceae	FR888536_f,Phophyromonadaceae, Lactobacillaceae, Lachnospiraceae
Udumula et al. [2]	NA	NA	NA	NA	NA	NA
Tanaka et al. [22]	NA	NA	NA	NA	NA	NA
Cortellino et al. [63]	NA	NA	NA	NA	NA	NA
Calderón-Montaño et al. [53]	NA	NA	NA	NA	NA	NA
Kuehm et al. [64]	NA	NA	NA	NA	Allobaculum, Clostridium, Parabacteroides, Lactococcus, Epulopiscium, Peptostreptococcaceae, Coriobacteriaceae	Rikenellaceae, RF39, Roseburia, Turicibacter, Anaeroplasma
Lam et al. [65]	NA	NA	NA	NA	Enterobacteriales, Verrucomicrobiales, Betaproteobacteriales	Firmicutes, Clostridiales and Lactobacillales
Li et al. [66]	NA	NA	NA	NA	Actinobacteria, Bifidobacterium longum, Olsenella spp	NA

*NA* not available

### Meta-analysis of fiber-high vs fiber-low diets

To provide a quantitative estimate of the association between fiber-rich dietary patterns and ICI response, we performed a limited random-effects meta-analysis using four prospective cohort studies that stratified patients into responders versus non-responders. For each study, we extracted or reconstructed 2 × 2 contingency tables comparing the most extreme diet categories (e.g., highest vs. lowest fiber or SCFA intake). Odds ratios (ORs) and 95% confidence intervals were calculated individually and pooled using a DerSimonian-Laird random-effects model. Notably, the data from Bolte et al. [19] were approximated based on reported response probabilities from logistic regression outputs at extreme Mediterranean diet adherence scores and were included to provide comparative context; these estimates should be interpreted cautiously due to the absence of raw group counts.

## Results

**Fig. 1:**
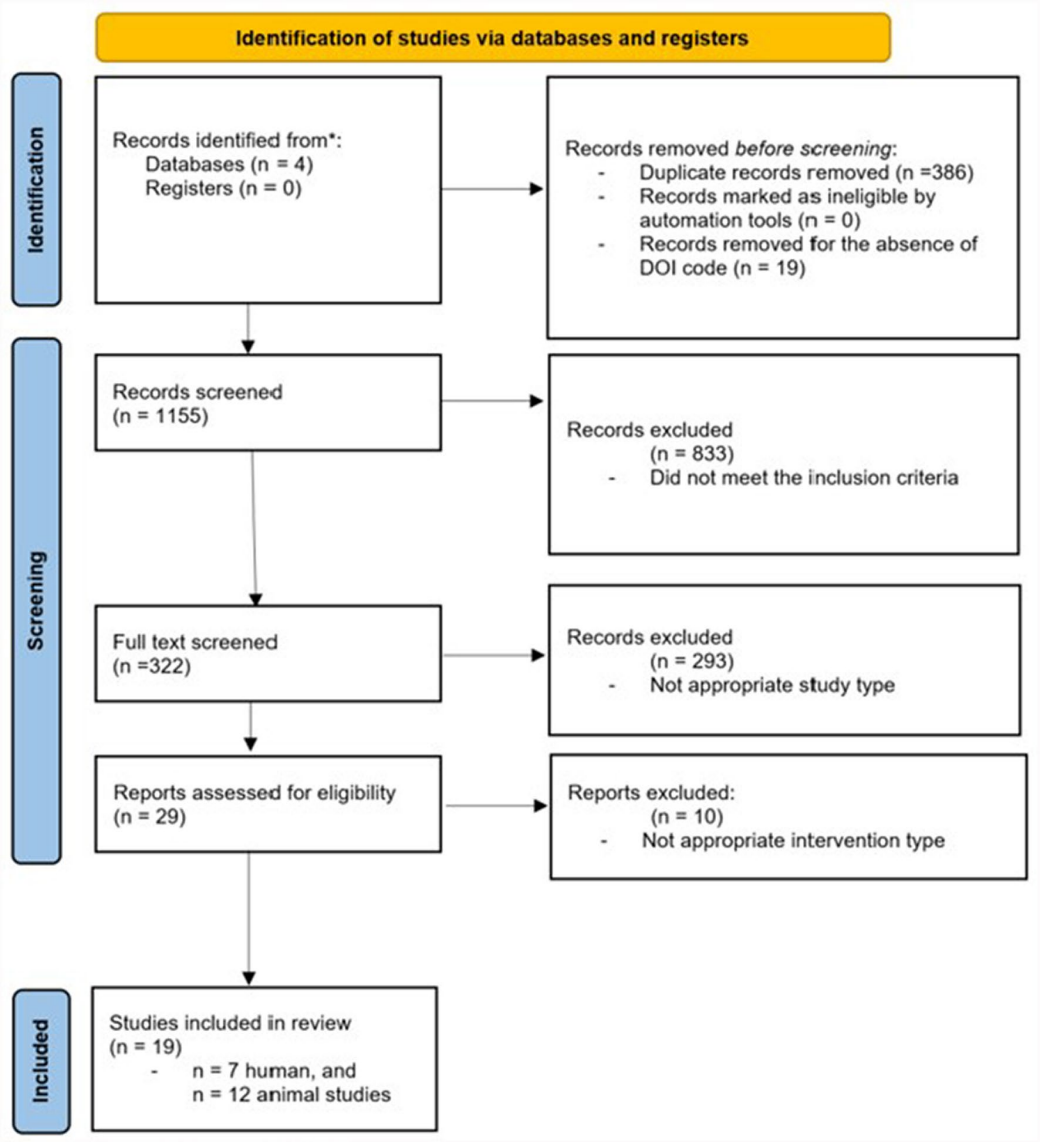
PRISMA flow chart of the used search methodology for identifying relevant papers. A systematic search of Web of Science, Medline, Cochrane Library, and Scopus was conducted to identify studies in which the immune function influential effect of dietary interventions were assessed in immune checkpoint inhibitor-treated mice and men. 19 studies underwent assessment based on predetermined eligibility criteria, with 10 being deemed unsuitable due to inappropriate intervention

A sum of 1155 research papers were evaluated in our comprehensive exploration of Medline, Web of Science, Scopus, and Cochrane Library databases. The procedure of selecting studies is illustrated in Fig. 1. Before the screening, 386 duplicated records were removed from the list, and another 19, due to the absence of the DOI code. Upon completion of the abstract screening process, 833 abstracts were deemed irrelevant and subsequently excluded. Afterwards, 322 studies underwent full-text screening, of which 293 were excluded for the same reason of irrelevance or not meeting inclusion and/or exclusion criteria. Of the 29 residual studies, 10 were disregarded due to the ineligibility of the applied intervention. Ultimately, 19 studies met the selection criteria and were incorporated into the systematic review.

### Quality assessment

The Newcastle–Ottawa scale (NOS) was employed to quantitatively evaluate the methodological quality of the 7 prospective cohort studies included. The evaluation was based on the Coding Manual for case–control and control studies, which assessed the study groups’ selection, comparability, and exposure/outcome aspects. None of the studies were identified as having a high risk of bias (0–5) according to the NOS criteria Supplementary Table 2.

**Fig. 2:**
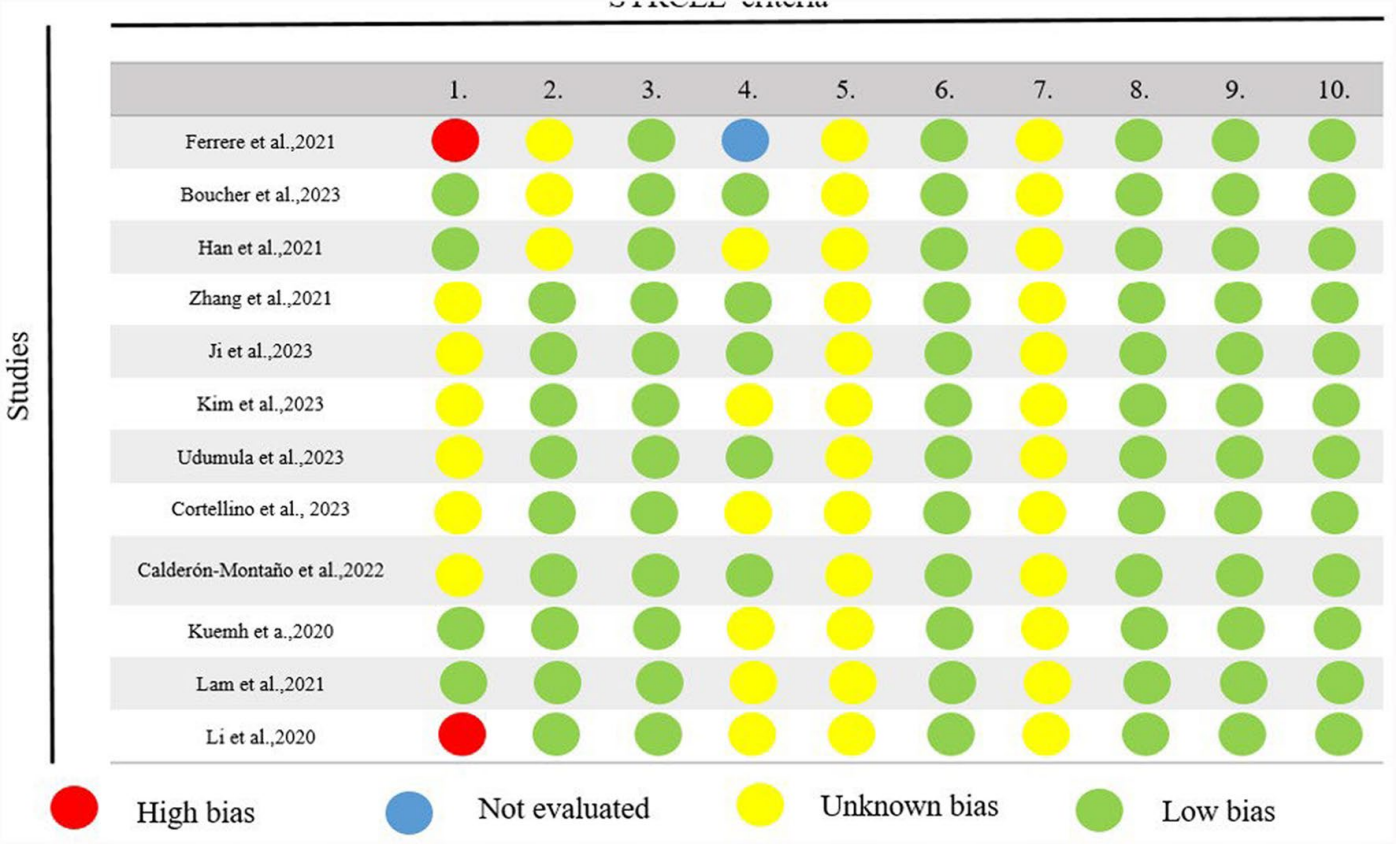
The potential for bias within the specific animal studies has been taken into consideration. The SYRCLE’s Risk of Bias tool was employed to evaluate the subsequent domains: 1, Was the allocation sequence adequately generated and applied? 2, Were the groups similar at baseline, or were they adjusted for confounders in the analysis? 3, Was the allocation to the different groups adequately concealed? 4, Were the animals randomly housed during the experiment? 5, Were the caregivers and/or investigators blinded from knowledge of which intervention each animal received during the experiment? 6, Were animals selected at random for outcome assessment? 7, Was the outcome assessor-blinded? 8, Were incomplete outcome data adequately addressed? 9, Are reports of the study free of selective outcome reporting? 10, Was the study apparently free of other problems that could result in high risk of bias? Abbreviations and symbols: Red circle: high bias, Blue circle: Not evaluated, Yellow circle: Unknown bias, Green circle: Low bias

The quality assessment of the animal studies was performed by the Systematic Review Center for Laboratory Aminal Experimentation (SYRCLE) risk of bias assessment tools. The findings regarding the attribution of bias across each domain of SYRCLE’s assessment tool are illustrated in Fig. 2. It is important to highlight that none of the studies sufficiently clarified whether proper blinding was applied regarding the caregivers’ or investigators’ awareness of the specific interventions administered to each animal throughout the research, or whether the outcome assessors were blinded or not. Also, all studies exhibited an absence of selective outcome reporting and did not manifest any additional discernible issues that might contribute to a heightened risk of bias.

### Study characteristics

The study characteristics are shown in Table 1. The used articles included 6 prospective cohort studies, and 1 observational cross-sectional study and 12 experimental randomized animal studies. The observed tumor types were melanoma (n = 6 human and n = 5 mouse), non-small cell lung cancer (NSCLC) in humans (n = 1), various types of solid tumors including head and neck cancer (n = 1), gastrointestinal cancer (n = 1), urothelial cancer (n = 1), glioma (n = 1), epithelial ovarian cancer (n = 2), colon carcinoma (n = 7 mouse), lymphoma (n = 1), renal cell carcinoma (n = 1 human and n = 1 mouse), and sarcoma (n = 1 human and n = 1 mouse). The applied tumor cell lines in mouse models are presented in Table 1. All of the study subjects were immune-checkpoint inhibitor (ICI) treated, only in the Pietrzak et al. [36] study observed 10 healthy individuals.

The study subjects (both humans and mice) were treated with anti-PD-1, anti-PDL-1, or anti-CTLA-4 inhibitors in monotherapy or combination therapy setup. In the case of human studies, information related to dietary habits were assessed by food frequency questionnaires. Some studies employed custom-made surveys, others used broadly accepted questionnaires to get more reliable and comparable results like EPIC-Norfolk FFQ [19] or National Cancer Institute Dietary Screener Questionnaire [20]. Food Frequency Questionnaire (FQ18N) developed by Wageningen University and Research, Cancer Council Victoria Dietary Questionnaire for Epidemiological Studies [21] and brief-type self-administered diet history questionnaire (BDHQ) [22]. From the surveys, calculations were made for macronutrient, micronutrient, and calorie consumption with a subsequent comparison of the findings between responders and non-responders, early or late responders, as well as long-term versus short-term survivors. The outcomes were reported in terms of overall response rate (ORR), progression-free survival (PFS), or overall survival (OS). In the case of pre-clinical studies, distinctions in microbial composition were evaluated through fecal sample sequencing, including 16S (n = 7) and shotgun (n = 2) and response assessment of tumor volume via caliper-based diameter measurements. Subcutaneous (n = 9) or orthotopic (n = 3) mice tumor models were used in the studies included in our systematic review.

High versus low fiber intake was observed in the relation of prolonged survival in the human studies. The alternate Mediterranean diet score (aMED), which measures adherence to a traditional Mediterranean diet marked by high consumption of vegetables, legumes, nuts, fruits, whole grains, and fish and low intake of red and processed meats, was used to evaluate dietary patterns in the study by Bolte et al. [19]. Furthermore, the percentage of plant-derived versus animal-derived foods was assessed using the original plant-based diet index (oPDI), which was further divided into two categories: the unhealthy plant-based diet index (u-PDI), which includes less nutrient-dense plant-based items like juices and refined grains, and the healthy plant-based diet index (h-PDI), which prioritizes unprocessed plant-based foods. Different thresholds have been used in different studies to determine high fiber consumption. While Simpson et al. [21] cited the Australian Dietary Guidelines, which suggest 30 g of fiber per day for males and 25 g per day for women, Pietrzak et al. [36] defined high fiber consumption as 20 g or more per day. Similarly, the criterion for a high fiber intake was set at 20 g per day by Spencer et al. [20]. Frequent use of foods high in fiber, such as green vegetables, cabbage, and mushrooms, was linked to higher levels of fecal short-chain fatty acids (SCFAs), according to Nomura et al. [34]. In the study by Golčić et al. [24], a high fiber intake was determined by 25 g daily, whereas a low intake is 22 g. Tanaka et al. [22], however, noted no significant difference in survival outcomes between individuals consuming 8.8 g per day and those consuming 11 g per day of fiber in both short- and long-term follow-ups. Table 4. lists Food frequency questionnaires (FFQs) used in human studies.

**Table 4:** Types of FFQs used in the included studies

Study	Questionnaire type
Bolte et al. [19]	EPIC-Norfolk FFQ
Pietrzak et al. [36]	Unique questionnaire evaluating fat, meat, fermented food, fruit, grain, salt, alcohol consumption, antibiotic, proton pump inhibitor usage
Simpson et al. [21]	Cancer Council Victoria Dietary Questionnaire for Epidemiological Studies (DQES v3.2) and Food Frequency Questionnaire (FQ18N) developed by Wageningen University and Research
Spencer et al. [20]	National Cancer Institute Dietary Screener Questionnaire
Nomura et al. [34]	Unique questionnaire evaluating beef, pork, chicken, fish, beans, green vegetables, cabbage, potato, radish, pumpkin, mushroom, seaweed, fruit, and yogurt consumption
Golčić et al. [24]	Croatian, Danish and American food composition database Danish and the Phenol-Explorer 3.0 database were used
Ferrere et al. [27]	Mouse study
Boucher et al. [25]	Mouse study
Han et al. [28]	Mouse study
Zhang et al. [1]	Mouse study
Ji et al. [52]	Mouse study
Kim et al. [45]	Mouse study
Udumula et al. [2]	Mouse study
Tanaka et al. [22]	Brief-type self-administered diet history questionnaire and DHQ Support Center software
Cortellino et al. [63]	Mouse study
Calderón-Montaño et al. [53]	Mouse study
Kuehm et al. [64]	Mouse study
Lam et al. [65]	Mouse study
Li et al. [66]	Mouse study

**Fig. 3:**
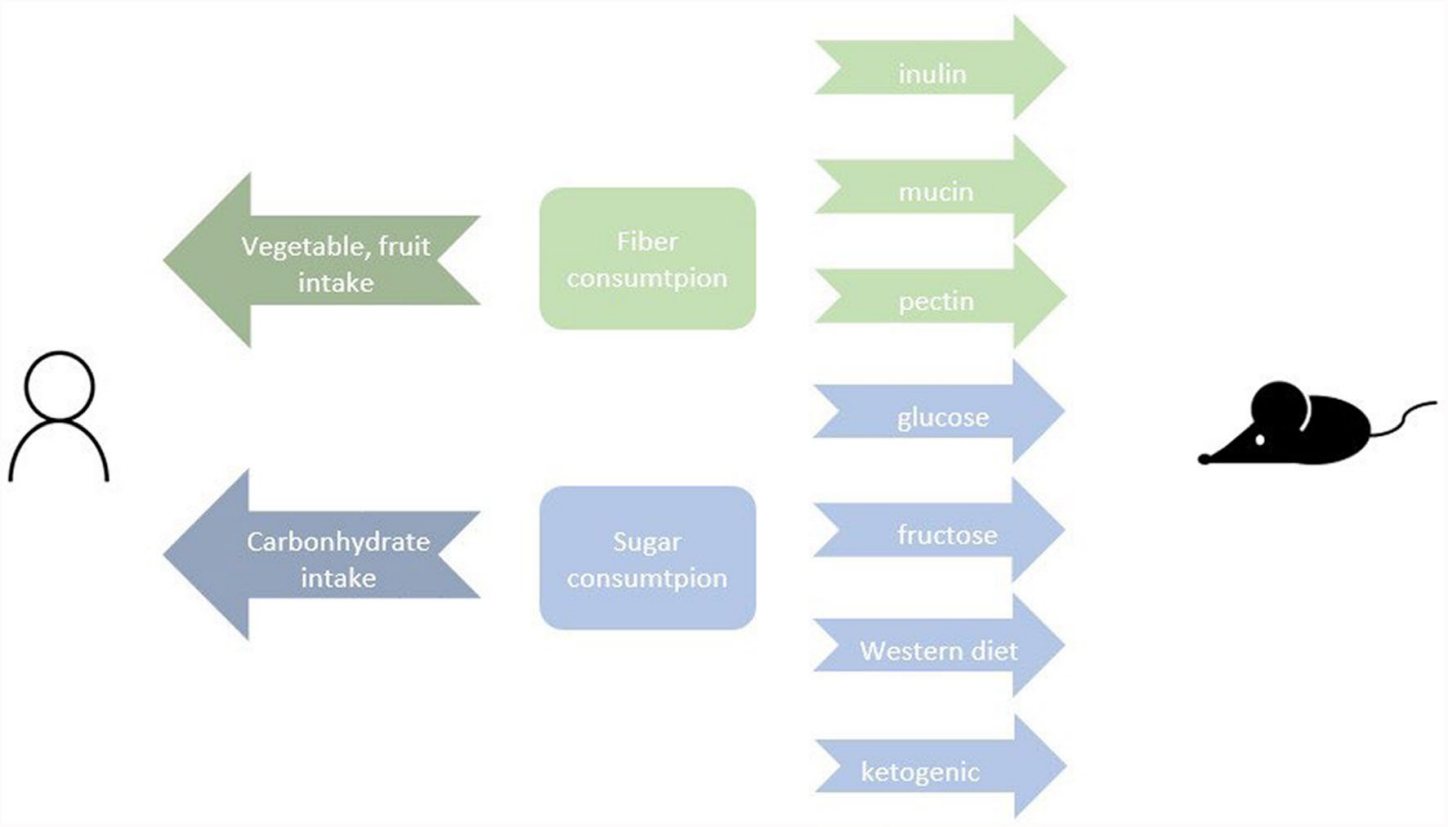
Associations of dietary interventions in mouse and human studies. Mouse studies highlight the underlying biology between specific dietary interventions and therapeutic responses to ICI. Fiber intake (marked with green) in human studies was presented in animal studies as inulin, mucin and pectin intake. Those mentioned molecules are prebiotics. Sugar consumption was categorized in human studies as carbohydrate intake, while in animal studies, the glucose, and fructose intake was observed as well as high sugar-, and fat-containing western type versus ketogenic diet

In mouse studies, various interventions were applied to reveal the response modifier effect of different dietary interventions. High fiber consumption was observed as inulin, pectin, and mucin supplementation which can have a similar effect as fruit and vegetable consumption in human patients [23]. High sugar intake was modeled as a high-glucose, fructose, and high sugar plus high fat-containing “western type” diet, which can be compared with ketogenic diet which is based on low carbohydrate consumption. The parallelized dietary interventions are shown in Fig. 3.

### Microbial taxonomy, diversity, and nutritional aspects

**Fig. 4:**
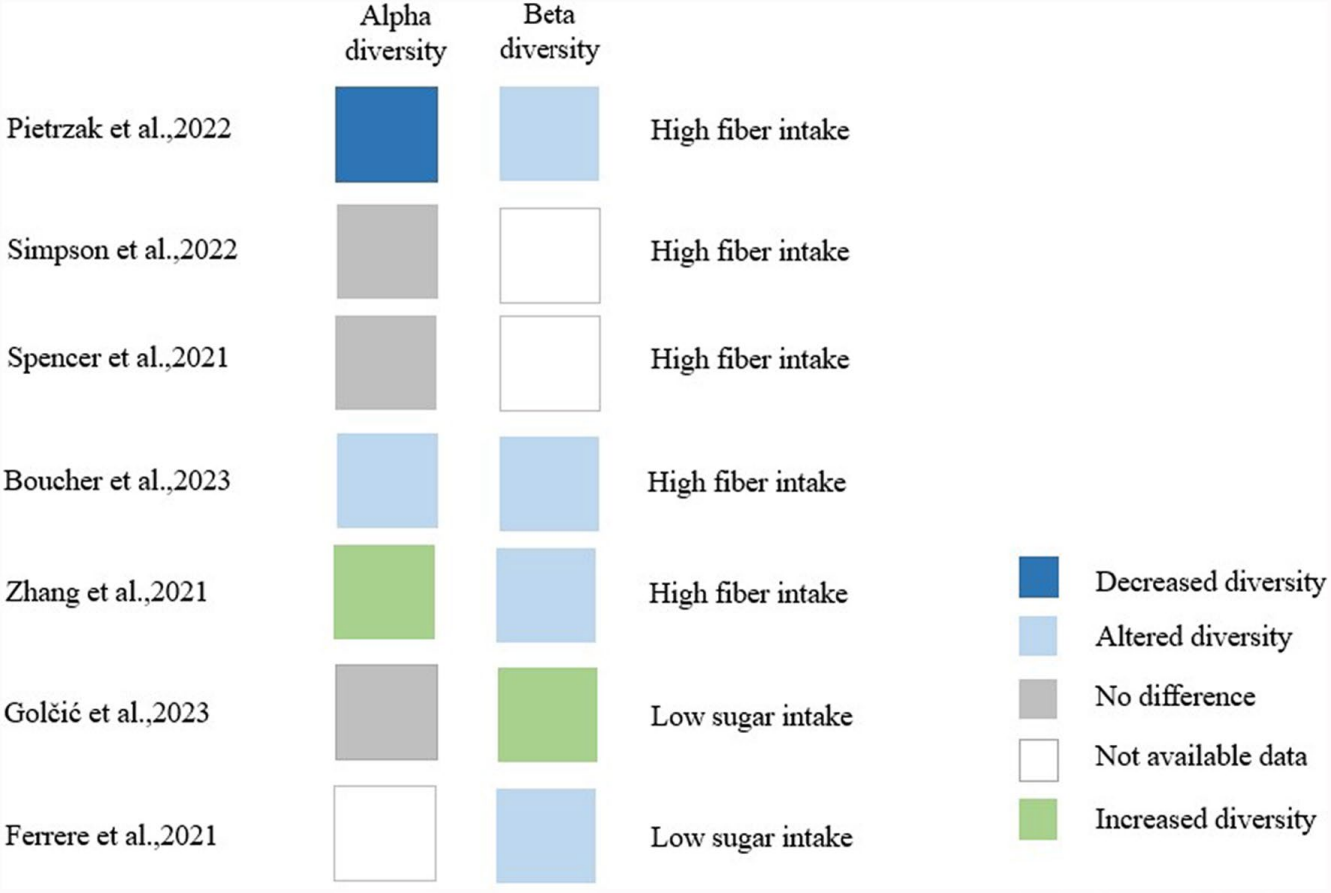
Bacterial diversity changes in patients with better outcomes after specific dietary interventions in the identified prospective cohort-, and observational cross-sectional studies

The overall alterations in the microbial communities were observed in 7 alpha and beta diversity studies (Fig. 4). Three human studies [20, 21, 24] did not find significant differences between alpha diversity of responder and non-responder patients, however Pietrzak et al. [36] reported significantly decreased alpha diversity in responders (Fig. 4). Beta diversity was reported to be increased in Golčić et al. [24]. The study evaluated both Jaccard distance and Bray–Curtis dissimilarity between the groups. It was observed in the study by Pietrzak et al. [36], that the beta diversity is significantly different (*p* = 0.003) defined by permutational multivariate analysis of variance in responder patients compared to non-responders. In animal studies, alpha, and beta diversity were reported after fiber supplementation [25, 26]. Altered beta diversity was observed following high fiber and ketogenic diet [25–27].

The article by Pietrzak et al. [36], and Simpson et al. [21]. described *Faecalibacterium prausnitzii* as an indicator of response, and they also highlighted the importance of a fiber-rich diet. In the study of Simpson et al. [21] *Akkermansia muciniphila* reported to be enriched in responders, as well as in a preclinical experiments, where mice were treated with a ketogenic diet [27], and inulin [28].

Observing the metabolic potential of the identified microbes in the context of therapeutic response, four SCFA producers and four lactic acid producer bacteria were identified as favorable. SCFA producers identified in responder patients were the *Faecalibacterium prausnitzii* [29]. *Bacteroides uniformis* [30] and *Butyricicoccus pullicaecorum* [1] species in addition to *Clostridium disporicum* [31]. An elevated presence of lactic acid producers were also reported, where *Ruthenibacterium lactatiformans* [32] after ketogenic diet, *Bifidobacterium animalis subsp. animalis* after inulin supplementation [33], and *Clostridium disporicum* [34] after inulin treatment were identified apart from the decreased abundance of *Turicibacter* [35] after high sugar and fat-containing nourishment.

### Comparison of the outcomes

The human studies used questionnaires to examine the nutritional differences according to response. In most of the human studies, PFS (n = 3) and ORR (n = 6) were assessed, the minority of studies (n = 2) observed the discrepancies between early and late responders. Besides response rate, n = 1 study evaluated the disease control rate (DCR).

In the studies by Bolte et al. [19] and Simpson et al. [21], a statistically significant distinction was observed between responders and non-responders (*p* = 0.02, False Discovery Rate [FDR]: 0.032) following high fiber consumption. Increased PFS was reported, with *p* = 0.03 and *p* = 0.01, respectively, linked to adequate (above median/mean) intake of fiber and mushrooms, as demonstrated by Spencer et al. [20] and Nomura et al. [34]. A substantial disparity was detected in the consumption of flavones (*p* = 0.027), and sugars (*p* = 0.04) in association with late versus early response, as reported in the study by Golčić et al. [24]. Likewise, a significant increase in responders was noted by Tanaka et al. [22] concerning increased seafood- (*p* = 0.045), and low sugar consumption (*p* = 0.031).

Bolte et al. [19] identified, that individuals with elevated fiber or unsaturated fat (characteristic of a Mediterranean dietary regimen) intake exhibited superior outcomes, with 46–49% attaining progression-free survival at the 12-month mark (PFS12) and 58–59% achieving objective response rates (ORR). Similarly, Pietrzak et al. [36] showed that individuals who responded favorably to ICI therapy consumed a higher fiber-containing diet, with reduced dairy intake, and elevated levels of SCFAs in their serum. Parallel to that, in the study by Nomura et al. [34], it was indicated, that patients with various cancers demonstrated higher fecal SCFA concentrations, associated with fiber intake, and also achieved a superior overall response rate (28.8%) and DCR (46.1%). After anti-PD-1 therapy, Simpson et al. [21] observed that melanoma patients exhibited favorable pathological responses, and had increased consumption of fiber and omega-3 fatty acids. In the study conducted by Spencer et al. [20] responder melanoma patients had elevated fiber intake, with 193 out of 293 patients (65.87%) achieving a favorable therapeutic outcome. Golčić et al. [24] showed, that individuals categorized as late responders to immunotherapy exhibited higher consumption of flavonoids, vitamin D, and anthocyanins, alongside reduced intake of sweets and saturated fats, thereby suggesting that particular nutrients may influence the timing and effectiveness of treatment. Tanaka et al. [22] revealed that long-term survivors (OS > 3 years) of NSCLC had increased consumption of seafood and eicosapentaenoic acid, which were positively correlated with increased OS.

**Fig. 5:**
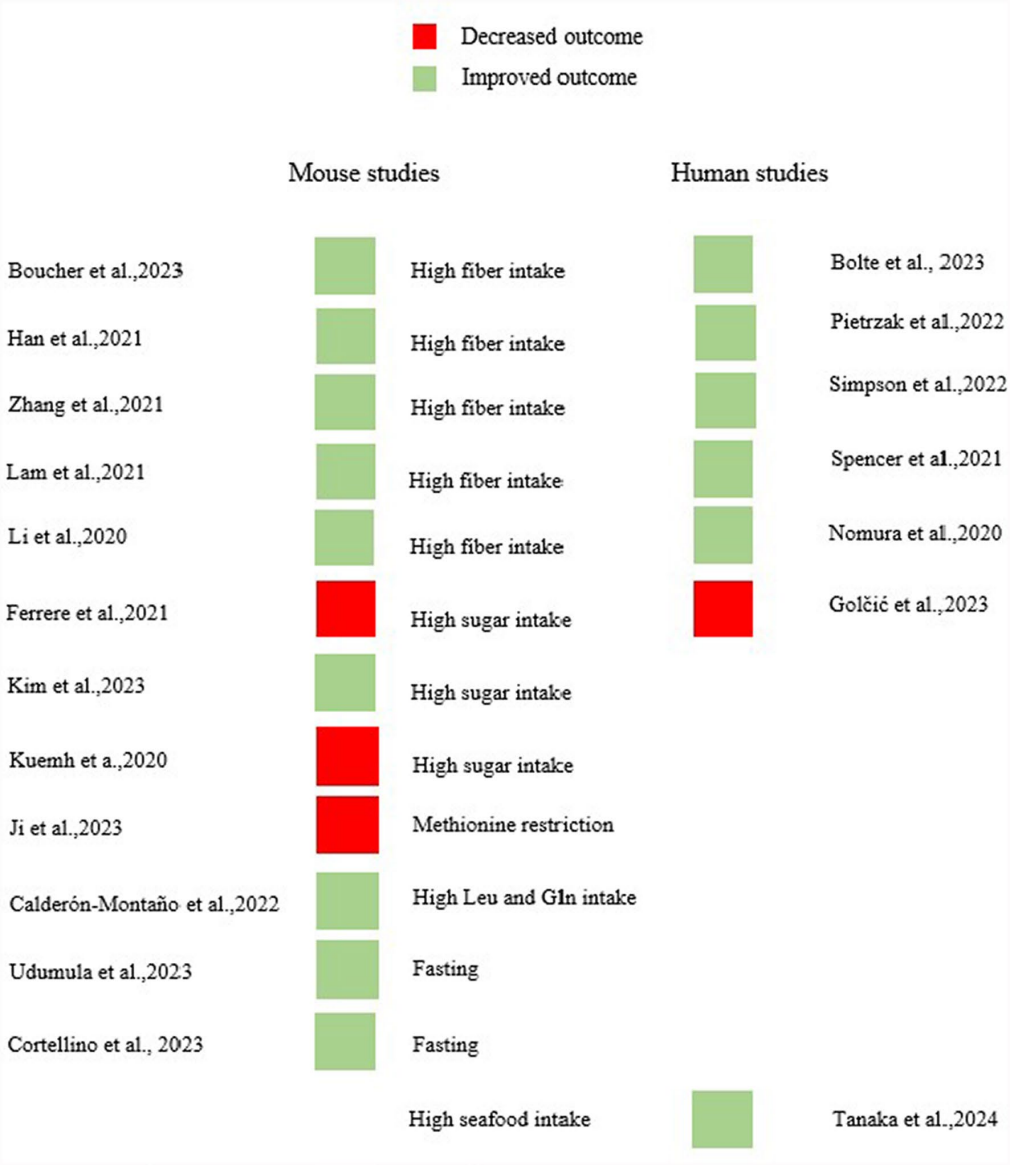
Associations of dietary habits and ICI efficiency in human and mouse data. Increased outcome in the association of a certain dietary habit was marked with green color. Decreased outcome with the association of a certain dietary habit was marked with red color

Across the entirety of the human studies, fiber, unsaturated fats, SCFA production, and specific nutrients such as omega-3 fatty acids and flavonoids were consistently associated with improved responses to immunotherapy, thereby improving survival (Fig. 5), indicating that dietary modifications could potentially play a pivotal role in augmenting treatment outcomes. The study by Ferrere et al. [27] revealed that adherence to a ketogenic diet markedly inhibited tumor proliferation, resulting in a significant decrease in tumor size and an extension of overall survival in 60% of mice injected with melanoma. Parallel to this, Kuehm et al. [64] documented that mice subjected to a high-glucose, Western-style diet exhibited diminished efficacy in immunotherapy and larger tumor sizes compared to control cohorts.

It was reported by Zhang et al. [1], that the intake of pectin substantially diminished tumor volume within a colorectal cancer model, thereby further substantiating the association between dietary fiber and enhanced outcomes after immunotherapy. In a similar vein, Boucher et al. [25], and Han et al. [28] showed that the supplementation of inulin decelerated tumor growth and also stimulated anti-tumor immune responses, with an inverse correlation observed between tumor size and the prevalence of advantageous microbiota such as *Akkermansia* and *Lactobacillus*. Calderón-Montaño et al. [53] demonstrated that particular restrictions on amino acids, safeguarded non-malignant cells and resulted in a diminished tumor burden and enhanced survival rates in mice receiving anti-PD1 immunotherapy. On the contrary, study by Ji et al. [52] reported that the dietary restriction of methionine resulted in increased colon tumor volumes.

In contrast, fasting-associated interventions consistently yielded beneficial results. Udumula et al. [2] documented significantly reduced tumor volumes in mice subjected to intermittent fasting, while Cortellino et al. [63] established that a diet, mimicking fasting-induced tumor regression, was characterized by a notable reduction in tumor size relative to standard chow-consuming mice. Collectively, these studies suggest that various dietary modifications can exert a potential influence on tumor growth (Fig. 5), with specific diets such as ketogenic, fasting, and high-fiber exhibiting considerable potential in reducing tumor size and augmenting the efficacy of immunotherapy. Patients with better survival exhibited longer PFS, and OS compared to patients with decreased survival. The unanimous finding of the studies comparing responder and non-responder patients is that the high amount of fiber consumption in the form of fruits and vegetables, as well as fish increased survival [19–21, 36]. It was also observed by Nomura et al. [34], that higher fecal SCFA content is associated with longer PFS, which can be a consequence of high fiber consumption. Observing the differences between long-term and short-term survivors, there was no detected difference in protein, fat, carbohydrate, fiber, and fatty acid intake overall, however long term-survivors consumed less sugar according to Tanaka et al. [22] and Golčić et al. [24]. 

### Quantitative synthesis of high-fiber diet impact on ICI response

We selected the four prospective human cohort studies for a partial meta-analysis, because they uniquely evaluated a clinically relevant binary outcome—response versus non-response to ICI—in relation to diet. Among the various dietary exposures assessed, the most comparable and clinically meaningful distinction across studies was the contrast between high versus low fiber intake. This dichotomy directly reflects dietary quality with known immunomodulatory potential via gut microbiota modulation. In addition to directly reported fiber intake [21, 36], two studies used established proxies for high fiber consumption: circulating short-chain fatty acid (SCFA) levels [34] and adherence to a Mediterranean diet [19], both of which reflect increased fiber intake and microbial fermentation. All included studies reported response outcomes in a form suitable for pooled analysis, enabling estimation of the effect size of fiber-rich versus fiber-poor diets across diverse populations and cancer types. The central research question addressed is whether high fiber intake—or its validated dietary or microbial surrogates—is consistently associated with improved ICI efficacy. This is clinically relevant as dietary modification is a feasible, low-cost, and scalable intervention that may improve immunotherapy outcomes and guide future interventional strategies.

**Fig. 6:**
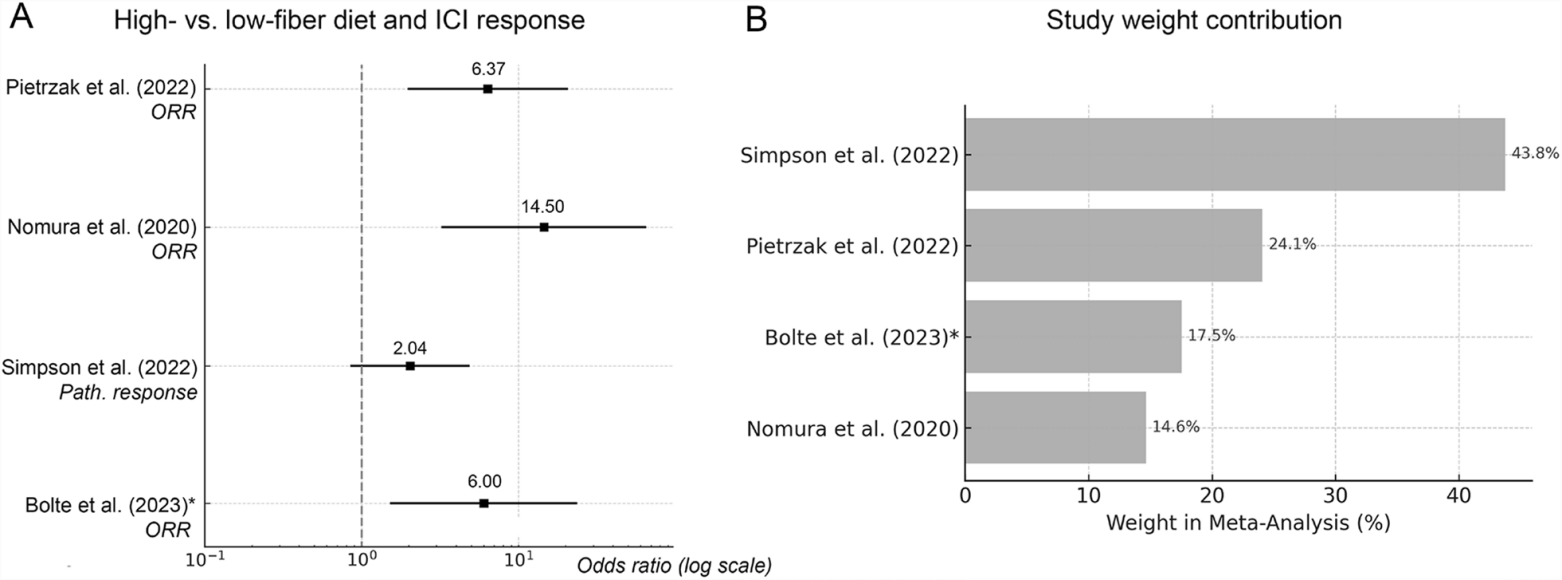
Meta-analysis of outcomes from prospective cohort studies. Forest plot showing odds ratios (log scale) for response to ICIs in patients with high- vs. low-fiber diets across four prospective studies. ORR = objective response rate; path. response = pathological response. **B** Relative study weights in the pooled random-effects meta-analysis based on inverse-variance estimation. Simulated odds from Bolte et al. [19] were included with caution due to estimated input values, contributing 17.5% to the overall summary effect

A random-effects meta-analysis was conducted using four prospective human studies that compared the likelihood of response to ICI between patients with fiber-rich or Mediterranean-like diets and those with fiber-poor diets (Fig. 6A). The most diet-extreme groups were selected from each study, and 2 × 2 contingency tables were constructed to calculate odds ratios (ORs) for responder versus non-responder outcomes. Data from *Bolte *et al*.* were simulated based on response probabilities due to lack of raw counts. The pooled analysis yielded a summary OR of 5.79 (95% CI: 2.45–13.68), indicating a significantly higher likelihood of ICI response among individuals adhering to fiber-rich diets. Study contributions to the summary effect were highest for Pietrzak et al. (32.5%) and Simpson et al. (28.4%), while Bolte et al., based on estimated data, contributed only 18.2% (Fig. 6B). Despite heterogeneity in dietary metrics and populations, all studies demonstrated directionally consistent results favoring dietary fiber as a supportive factor for ICI efficacy.

## Discussion

Recently, anti-cancer ICI therapy outcomes have been linked to microbiota [37, 38]. Nutritional habits might influence the gut microbiome, which holds the potential to alter taxa abundance, diversity, and functionality. The advantageous impacts of consuming low sugar or ketogenic diet along with high dietary fiber arise from its physical, immunomodulatory, and prebiotic characteristics [39, 40]. These benefits are commonly linked to the increased production of SCFAs, which play a pivotal role in the modulation of immunological tolerance through both lymphocytes and myeloid cells and by decreasing tumor necrosis factor (TNF)-α, interferon (IFN)-γ, interleukin (IL)-17,10,6 and IL13 production. Short-chain fatty acids (SCFAs) modulate immune tolerance by influencing lymphocyte and myeloid cell function and reducing pro-inflammatory cytokines such as TNF-α, IFN-γ, IL-17, IL-10, IL-6, and IL-13 [41]. SCFAs impact chemotaxis, phagocytosis, ROS production, cellular proliferation, and have anti-inflammatory, antitumorigenic, and antimicrobial properties. They enhance epithelial integrity [46], activate the NLRP3 inflammasome to induce IL-18 secretion, promote neutrophil recruitment, and support FOXP3+ regulatory T cell differentiation. Butyrate acts via G protein-coupled receptor activation and histone deacetylase inhibition, increasing tight junction and antimicrobial peptide expression [47]. SCFA producers function as significant agents in the conservation of gastrointestinal homeostasis through the enhancement of epithelial integrity and the improvement of mucosal immunity, whereas current investigations suggest that they are also indispensable for the regulation of systemic immunity [41].

In the present systematic review, we aimed to summarize the current evidence on clinical and preclinical data on nutritional habits, gut microbiota taxonomy, diversity, and ICI treatment outcomes. The majority of studies showed that fiber-rich diet associated with favorable outcomes in ICI-treated patients [42, 43, 45]. The observed human studies [19–22, 36] in agreement, also highlighted the importance and beneficial effects of fruit and vegetable consumption, Nomura et al. [34] also emphasizing the significant beneficial effect of mushroom intake in responders. However, Russo et al. [43] reported increased bacterial diversity parallel to fiber consumption, Pietrzak et al. [36] observed decreased alpha-, and altered beta-diversity in responders in the case of elevanted fiber consumption. Analyzing the results of precilincal mouse studies, Ferrere et al. [27], Boucher et al. [25] and Zhang et al. [1] reported altered beta-diversity after ketogenic, and fiber-enriched diet. Following high fiber-containing diet, Zhang et al. [1] and Boucher et al. [25] reported increased and altered alpha-diversity. The identified results underpin the hypothesis of the correlation between high-fiber diet and beneficial effect on ICI outcome. Furthermore, the quantitative synthesis suggested that high fiber intake—or its validated dietary and microbial proxies—was consistently associated with improved response to ICIs. The pooled effect estimate (OR = 5.79) and directionally aligned results across studies shows the clinical relevance of diet as a modifiable factor in immunotherapy outcomes. Despite some heterogeneity and one study relying on estimated data, the findings support dietary fiber as a low-cost, scalable strategy to enhance ICI efficacy and justify prospective interventional trials.

Cancer alters the body’s utilization of amino acids. Tumors rely on amino acids as a fuel source and source of antioxidants to regulate the generation of harmful reactive oxygen species. Amino acids play a significant role in modifying the epigenetic information to either upregulate or downregulate the activity of genes associated with tumors [46]. Dietary addition or restriction of certain amino acids were examined by several studies [47–51]. In the included studies, the impact of restricting dietary methionine [52] and the influence of changing the amino acid composition of the diet [53] were also investigated. The immunostimulatory effect of methionine in immunocompetent mice was evidenced by the production of H2S by microbes according to Ji et al. [52] and Calderón-Montaño et al. [53]. The Authors reported, that methionine restriction alone is not sufficient to enhance anti-cancer activity, the composition of other amino acids and nutrients are also important. Altogether, amino acid composition of diet has a significant effect on anti-cancer immunity, in which processes intestinal microbiome has a crucial role.

Epidemiological data has continuously established a correlation between sugar consumption with elevated occurrences of obesity, metabolic syndrome, and diabetes, all of which represent predisposing factors for cancer [44]. In a colorectal cancer model, high sugar diet exhibited a promotion of tumor growth, despite the absence of metabolic alterations like diabetes [45]. In the study by Golčić et al. [24], it was observed, that patients who exhibited complete remission consumed smaller amounts of sweets. The detrimental effect of elevated sugar intake was confirmed by Kuehm et al. [64] by observing that Western-diet impaired the response to ICI therapy. Microbial differences between Western-diet and normal groups were also identified. These results are also supported by Ferrere et al. [27], namely that the ketogenic diet led to alterations in the composition of the gut microbiota, in both mice and humans following carbohydrate-restricted dietary interventions resulting in enhanced anti-neoplastic effect of T cells in the tumor microenvironment. In contrast, Kim et al. [45] observed that short-term high glucose supplementation enhanced anti-tumor immunity in a glioblastoma multiforme (GBM) mouse model. There is a difference in the tumor microenvironment between immunologically “hot” tumors, like melanoma, colon cancer, NSCLC, and “cold” tumors like GBM, which can have an impact of the different behavior of the mentioned tumor after glucose supplementation [45].

Modulating the gut microbiota is a promising strategy to enhance the efficacy of immune checkpoint inhibitors (ICIs), with three primary approaches under investigation: fecal microbiota transplantation (FMT), probiotics, and dietary interventions. While probiotics have shown potential in preclinical and observational studies, clinical trials have yielded inconclusive results, and non-personalized use may reduce microbial diversity, potentially impairing ICI outcomes [9, 12]. FMT, by contrast, has demonstrated clinical benefit in refractory cancers by increasing microbial diversity and enriching beneficial taxa associated with improved immunotherapeutic responses [54] However, due to its invasiveness, dietary interventions offer a more practical, non-invasive alternative. Diets rich in fiber and low in sugar may mimic the microbiota-modulating effects of FMT and enhance ICI efficacy [9, 57]. Reflecting this potential, several clinical trials (NCT04645680, NCT05356182, NCT06236360, NCT04866810, NCT06671613) are evaluating dietary strategies, particularly in non-small cell lung cancer and melanoma. Although most remain ongoing, the increasing focus on dietary modulation underscores its appeal due to its safety, cost-effectiveness, and accessibility for improving immune responses in oncology patients.

Another conclusion from studies in the field is that fasting and ketogenic diets may offer immunotherapeutic benefits, their application in cancer patients—especially those with cachexia or treatment-related side effects—requires caution [39, 55]. Cachexia, characterized by severe weight and muscle loss, may be worsened by restrictive diets. Arends and colleagues emphasize the importance of nutritional support and advise against further nutrient restriction in malnourished patients [56]. Adherence to intermittent fasting or ketogenic diets is also challenged by appetite loss and treatment side effects. Klement and colleagues highlight the potential of ketogenic diets in oncology but note adherence difficulties due to their restrictive nature [57]. These findings are also in line with recent perspectives on the interplay between diet, the microbiome, and immune modulation in cancer [58, 59]. Future studies should evaluate the long-term safety, metabolic effects, and feasibility of such diets in cancer patients and explore personalized dietary strategies balancing efficacy with nutritional adequacy.

Diverse food FFQs introduce systematic measurement bias, often leading to over- or underestimation of dietary intake and reduced cross-study comparability. Self-reported data are prone to response and social desirability bias, increasing misclassification and compromising dietary exposure precision [60, 61]. Errors also arise from inaccurate portion size estimation and recall difficulties. Differences in FFQ design and validation across populations further limit accuracy when applied to diverse cohorts. These limitations challenge FFQs’ ability to detect diet–disease associations, raising concerns about their reliability in epidemiological research [62].

The included studies had varying primary scopes: human studies predominantly examined fecal microbiome differences between ICI responders and non-responders or assessed serum fatty acid content in blood plasma. Dietary information obtained via food frequency questionnaires was secondary scope to these main focuses. In addition, differences in FFQs introduce measurement errors, response bias, portion size variability, and population-specific inaccuracies, potentially limiting their reliability in detecting diet-disease associations, and cross-study comparison. Additionally, outcomes were often evaluated and reported as categorical rather than continuous variables, limiting data comparison or detailed analysis. Mouse studies varied in dietary interventions; the caregivers and/or investigators were not blinded, nor were animals selected at random for outcome assessment, making challenging comparisons across studies. More research is needed, specifically on how distinct dietary interventions influence immune checkpoint inhibitor (ICI) efficacy. 

## Conclusions

Intermittent fasting, high fiber, and low sugar consumption are significantly associated with better ICI outcomes. Clinical prospective cohort studies revealed alterations in microbiota composition linked to diet. The alterations in microbiota composition linked to diet appear to be confirmed in animal models, particularly regarding the production of SCFAs and lactic acid, as well as an increase in Firmicutes/Bacteroidetes ratio modifications in the overall microbial diversity. Further prospective studies are needed to accelerate microbiota therapeutics in the field. 

## Supplementary Information

Additional file 1.

Additional file 2.

## Data Availability

This was a systematic review, where no data were generated or deposited by the authors. All data is accessible in the included articles.

## Abbreviations

CHT: Chemotherapy
CRT: Chemoradiotherapy
IO: Immuno-oncology
IT: Immunotherapy
ICI: Immune checkpoint inhibitors
SCFA: Short-chain fatty acid
F/B ratio: Firmicutes/ Bacteroidetes ratio
NA: Not assessed
PFS: Progression-free survival
ORR: Overall response rate
PFS12: PFS at 12 months
RECIST: Response evaluation criteria
NSCLC: Non-small cell lung cancer
GMB: Glioblastoma multiforme
TNF-α: Tumor necrosis factor
IFN-γ: Interferon gamma
DCR: Disease control rate
