# Supplementary material for: Gut microbiome changes and cancer immunotherapy outcomes associated with dietary interventions: a systematic review of preclinical and clinical evidence
Source: J Transl Med. 2025 Jul 8;23:756. doi: 10.1186/s12967-025-06586-0 (PMC12239337; doi:10.1186/s12967-025-06586-0)
Supplement: Supplementary file 1 — Additional file 1. [file 12967_2025_6586_MOESM1_ESM.docx]

**NEWCASTLE-OTTAWA scale**

**CASE-CONTROL/COHORT STUDIES**

| **FIRST AUTHOR** | **YEAR** | **SELECTION** | **COMPARIBILITY** | **EXPOSURE/OUTCOME** | **SCORE** |
| --- | --- | --- | --- | --- | --- |
| Bolte | 2023 | ★★★ | ★★ | ★★★ | 8 |
| Pietrzak | 2022 | ★★ | ★★ | ★★★ | 7 |
| Simpson | 2022 | ★★★★ | ★★ | ★★★ | 9 |
| Spencer | 2021 | ★★★★ | ★★ | ★★★ | 9 |
| Nomura | 2020 | ★★★ | ★★ | ★★★ | 8 |
| Golčić | 2023 | ★★ | ★★ | ★★★ | 7 |
| Tanaka | 2024 | ★★ | ★★ | ★★★ | 7 |
