# Supplementary material for: Gut microbiome changes and cancer immunotherapy outcomes associated with dietary interventions: a systematic review of preclinical and clinical evidence
Source: J Transl Med. 2025 Jul 8;23:756. doi: 10.1186/s12967-025-06586-0 (PMC12239337; doi:10.1186/s12967-025-06586-0)
Supplement: Supplementary file 2 — Additional file 2. [file 12967_2025_6586_MOESM2_ESM.docx]

Supplementary file 1.

Web of Science search key:

Web of Science: AB=((Diet* OR Vegetable OR Fruit OR Meat) AND (Cancer OR Tumor* OR Tumour*) AND (Metagenom* OR 16S* OR Microbiot* OR Microbiome OR Commensal* OR Flora) AND (Gut OR Intestinal* OR Gastrointestinal*) AND (Radiotherap* OR Chemoradiotherapy OR Chemoradiation OR Chemotherap* OR Targeted therap* OR immunotherapy OR ICI OR immune checkpoint)) n=295, from this 169 written in english

Cohrane Library search key:

(Diet\ *OR Vegetable OR Fruit OR Meat) AND (Cancer OR Tumor* OR Tumour*) AND (Metagenom* OR 16S\ *OR Microbiot* OR Microbiome OR Commensal\ *OR Flora) AND (Gut OR Intestinal* OR Gastrointestinal*) AND (Radiotherap* OR Chemoradiotherapy OR Chemoradiation OR Chemotherap\ *OR Targeted therap* OR immunotherapy OR ICI OR immune checkpoint) n=35

Scopus search key:

( TITLE-ABS ( diet* OR vegetable OR fruit OR meat ) AND ( cancer OR tumor* OR tumour* ) AND ( metagenom* OR 16s* OR microbiot* OR microbiome OR commensal* OR flora ) AND ( gut OR intestinal* OR gastrointestinal* ) AND ( radiotherap* OR chemoradiotherapy OR chemoradiation OR chemotherap* OR targeted AND therap* OR immunotherapy OR ici OR immune AND checkpoint ) ) AND ( LIMIT-TO ( LANGUAGE , "English" ) ) n=706 written in english

Medline search key:

(Diet* OR Vegetable OR Fruit OR Meat) AND (Cancer OR Tumor* OR Tumour*) AND (Metagenom* OR 16S* OR Microbiot* OR Microbiome OR Commensal* OR Flora) AND (Gut OR Intestinal* OR Gastrointestinal*) AND (Radiotherap* OR Chemoradiotherapy OR Chemoradiation OR Chemotherap* OR Targeted therap* OR immunotherapy OR ICI OR immune checkpoint), n=447
